# Histological evaluation in ulcerative colitis

**DOI:** 10.1093/gastro/gou031

**Published:** 2014-06-18

**Authors:** Tom C. DeRoche, Shu-Yuan Xiao, Xiuli Liu

**Affiliations:** ^1^Department of Pathology, Wake Forest Baptist Medical Center, Winston-Salem, North Carolina, USA; ^2^Department of Pathology, University of Chicago, Chicago, Illinois, USA; ^3^Department of Anatomic Pathology, Cleveland Clinic, Cleveland, Ohio, USA

**Keywords:** ulcerative colitis, histological evaluation, dysplasia, differential diagnosis

## Abstract

This review summarizes diagnostic problems, challenges and advances in ulcerative colitis (UC). It emphasizes that, although histopathological examination plays a major role in the diagnosis and management of UC, it should always be interpreted in the context of clinical, endoscopic, and radiological findings. Accurate diagnosis requires knowledge of the classic morphological features of UC, as well as a number of atypical pathological presentations that may cause mis-classification of the disease process, either in resection or biopsy specimens. These atypical pathological presentations include rectal sparing and patchiness of disease at initial presentation of UC in pediatric patients or in the setting of medically treated UC, cecal or ascending colon inflammation in left-sided UC, and backwash ileitis in patients with severe ulcerative pancolitis. Loosely formed microgranulomas, with pale foamy histiocytes adjacent to a damaged crypt or eroded surface, should not be interpreted as evidence of Crohn’s disease. Indeterminate colitis should only be used in colectomy specimens as a provisional pathological diagnosis. Patients with UC are at risk for the development of dysplasia and carcinoma; optimal outcomes in UC surveillance programs require familiarity with the diagnostic criteria and challenges relating to UC-associated dysplasia and malignancy. Colon biopsy from UC patients should always be evaluated for dysplasia based on cytological and architectural abnormalities. Accurate interpretation and classification of dysplasia in colon biopsy from UC patients as sporadic adenoma or UC-related dysplasia [flat, adenoma-like, or dysplasia-associated lesion or mass (DALM)] requires clinical and endoscopic correlation. Isolated polypoid dysplastic lesions are considered to be sporadic adenoma if occurring outside areas of histologically proven colitis, or adenoma-like dysplasia if occurring in the diseased segment. Recent data suggest that such lesions may be treated adequately by polypectomy in the absence of flat dysplasia in the patient. UC patients with DALM or flat high-grade dysplasia should be treated by colectomy because of the high probability of adenocarcinoma. The natural history of low-grade dysplasia (LGD) is more controversial: while multifocal LGD, particularly if detected at the time of initial endoscopic examination, is treated with colectomy, unifocal flat LGD detected during surveillance may be managed by close follow-up with increased surveillance. The surveillance interval and treatment options for UC patients with dysplasia are reviewed in detail.

## INTRODUCTION

Histopathological examination plays a major role in the diagnosis and management of ulcerative colitis (UC), but should always be interpreted in the context of clinical, endoscopic, and radiological findings. Accurate diagnosis requires knowledge of the classic morphological features of UC, as well as a number of atypical pathological presentations that may cause mis-classification of the disease process. Optimal outcomes in UC surveillance programs require familiarity with the diagnostic criteria and challenges relating to UC-associated dysplasia and malignancy. We provide a brief overview of the pathological features and differential diagnosis of UC, the concept of indeterminate colitis, and neoplastic complications of UC.

## MACROSCOPIC FEATURES

**Figure 1.**
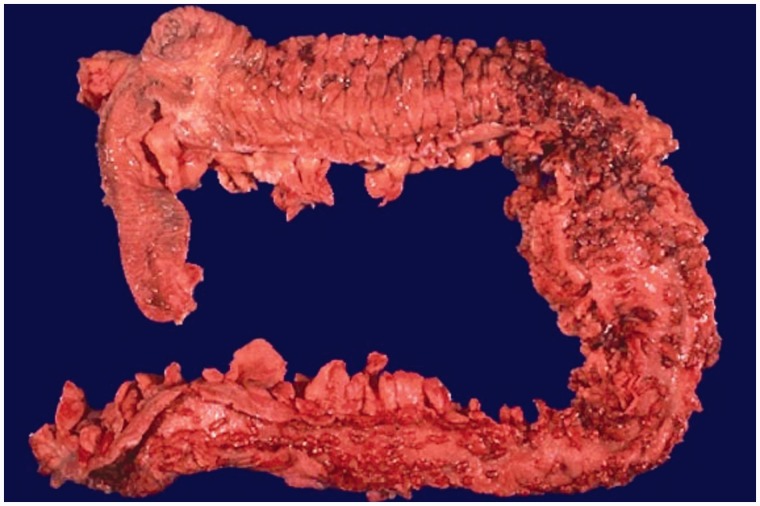
Gross photograph of ulcerative colitis. Diffuse erythema, edema, and many inflammatory polyps are noted in the rectum, left colon, transverse colon, and hepatic flexure. The right colon and terminal ileum are normal.

UC is characterized by diffuse (contiguous and symmetrical) inflammation, restricted to the colonic mucosa. Overall, the resected specimen will show diffuse mucosal granularity, edema, and erythema with or without ulceration (Figure 1). These mucosal changes involve the rectum and variable lengths of the proximal colon in continuity; the distal colon should be more severely diseased than the proximal colon. According to the extent of disease, UC can be divided into ulcerative proctitis, left-side colitis, sub-total colitis, and pancolitis [1]. The bowel wall maintains its normal thickness, reflecting the absence of transmural inflammation, and no fat wrapping, strictures, or fistula tracts should be present. In long-standing and severe cases, there may be ‘cobblestoning' and slight but diffuse narrowing of the distal colon. Mucosal polyps of sessile, pedunculated, or filiform configuration may also be seen in long-standing disease.

## MICROSCOPIC FEATURES

### Features of classic cases

**Figure 2.**
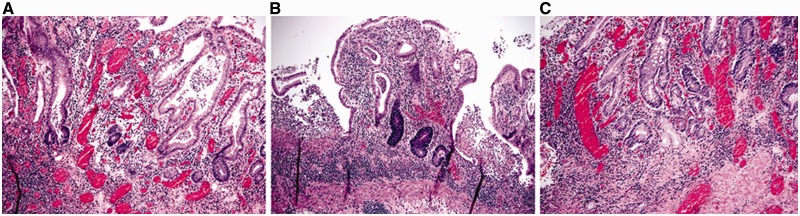
Microscopic features of ulcerative colitis. A and B: architectural distortion, including shortening of crypts, variation in the sizes and shapes of crypts, and basal lymphoplasmacytosis (A & B: H&E stain; 100X). C: Paneth cell metaplasia and pyloric gland metaplasia in the left colon (H&E stain; 100X).

In untreated disease, UC usually exhibits a histological pattern of chronic active colitis, which refers to the presence of active inflammation accompanied by features of chronic mucosal injury. Activity is defined as the presence of neutrophil-mediated epithelial injury, which may take the form of neutrophils infiltrating crypt epithelium (cryptitis), collections of neutrophils within crypt lumens (crypt abscesses), or by infiltration of surface epithelium with or without mucosal ulceration. Chronicity is defined by crypt architectural distortion, basal lymphoplasmacytosis, or Paneth cell metaplasia in the left colon. Architectural distortion is represented by shortening of the crypts (i.e. presence of space between the bottom of the crypts and the upper edge of the *muscularis mucosae*) and branching of the crypts (Figures 2A and B). In normal mucosa, the crypts are uniformly spaced, arranged perpendicular to the *muscularis mucosae*, and the crypt bases contact the upper edge of the *muscularis mucosae*. Basal lymphoplasmacytosis refers to the presence of a lymphoplasmacytic infiltrate between crypt bases and the *muscularis mucosae* (Figure 2B). While Paneth cells are a normal component of the right colon, their presence in the left colon is a metaplastic process, due to chronic crypt epithelial injury (Figure 2C). Rarely, pyloric gland metaplasia may also be seen in UC (Figure 2C). Microscopically, these changes of chronic active colitis are diffuse and uniform in distribution: that is, every biopsy fragment from the diseased colon shows a similar degree of injury and inflammation.

The severity of UC is primarily determined clinically, on the basis of symptoms, findings on physical examination, and laboratory tests. Histological assessment of the severity of UC is often expected by the treating clinician and may be attempted by pathologists, though this practicing pattern varies greatly. Histological quantification of the degree of inflammation is a rough estimate due to limited sampling, and probably lacks reproducibility. Nevertheless, histological quantification of disease activity might be useful, as the histological severity of inflammation over time is a risk factor for colo-rectal neoplasia in UC [2]. Semi-quantitative criteria for histological assessment of inflammation have been proposed [2]. In this scheme, mild cases show expansion of the *lamina propria* by lymphocytes and plasma cells, with infiltration of surface/crypt epithelium by neutrophils and/or crypt abscesses present in less than 50% of crypts. In moderately active cases, cryptitis and crypt abscesses involve more than 50% of crypts. Severely active cases are defined by the presence of erosion or ulceration.

With spontaneous healing or medical treatment, UC may become inactive or quiescent. Histologically, inactive (quiescent) colitis is characterized by marked architectural abnormalities in the absence of active inflammation, and the most commonly observed architectural abnormalities include atrophy, irregularity and shortening of crypts, thickening of the *muscularis mucosae*, and metaplasia (Paneth cell metaplasia in the left colon, or pyloric-type glands in any location).

### Evaluation of initial colo-rectal biopsies prior to medical treatment

Evaluation of the first set of colo-rectal biopsies prior to medical treatment plays an essential role in the management of patients with acute onset bloody diarrhea. In this clinical setting, the primary differential diagnostic considerations are acute infectious colitis and idiopathic inflammatory bowel disease (IBD) (including UC). Both of these entities invariably show active disease in the form of neutrophilic epithelial injury. However, a reliable criterion in distinguishing UC from acute infectious colitis is the presence of histological features of chronicity [3]. Even in acute presentations of UC, well-developed features of chronicity (including architectural distortion, basal lymphoplasmacytosis, or left-sided Paneth cell metaplasia) should be present.

It is not routine practice to definitively diagnose UC *vs* Crohn’s disease in the initial biopsy. Given the superficial nature of endoscopic biopsy, one of the criteria for distinguishing UC *vs* Crohn’s disease (namely superficial *vs* transmural inflammation) cannot be assessed. As will be discussed later, there are a number of atypical pathological features that may lead to misclassification of IBD, especially in biopsy specimens. Nevertheless, assessment of disease distribution in the colon may be helpful in assisting to distinguish UC from Crohn’s disease; the best specimen for this purpose is the first set of ‘systematic' biopsies prior to medical treatment. The key histological features supporting a diagnosis of UC in pre-treatment biopsies include a diffuse and uniform chronic active colitis with a universal involvement of the rectum and absence of granulomas.

### Atypical features of UC prior to medical treatment

Atypical features may be observed in pre-treatment biopsies in patients with clinically proven UC. The presence of these features may raise concerns regarding the true nature of the underlying colitis and may lead to an erroneous diagnosis of Crohn's disease. An awareness of these atypical features and their associated clinical settings is essential, to avoid mis-diagnosis.

#### Pre-treatment presentation of UC in children

Several studies have shown that endoscopic or histological rectal sparing (either relative or absolute) may occur at the time of initial presentation in a small subset of pediatric UC patients [4–7]. In the largest study by Glickman *et al.* [7], relative rectal sparing (defined as less-severe inflammation in the rectum, compared with the more proximal colon) or absolute rectal sparing (meaning normal histology in the rectum) occurs in 30% of pediatric UC cases. Similarly, patchy inflammation is noted in 21% of pediatric UC cases [7].

#### Cecal or ascending inflammation in left-sided UC

Some patients with left-sided UC may have inflammation in the cecum and/or ascending colon, but with complete endoscopic and histological sparing of the intervening transverse colon [8–12]. Mutinga *et al.* (2004) have found that patchy right-sided inflammation was present in 9% of unselected UC patients with predominantly left-sided disease [11]. Therefore, in patients with left-sided colitis, inflammation in the cecum or ascending colon with sparing of the transverse colon should not be construed as a skip lesion favoring Crohn's disease.

#### Fulminant UC

Patients with fulminant UC may show some Crohn's-like features, such as rectal sparing and linear deep ulcerations, which may lead to an erroneous diagnosis of Crohn's disease on mucosal biopsy evaluation [13]. Knowledge of the clinical presentation may be helpful in avoiding misdiagnosing such cases as Crohn's disease.

#### Backwash ileitisin UC

Inflammation of the terminal ileum (up to a few centimeters) is reported to occur in about 17% of UC patients [14]. However, there are no widely accepted diagnostic criteria for backwash ileitis [14–16]. Detailed histological study of colectomies from UC patients reveals that most backwash ileitis cases also had severe inflammatory activity in the colon (65%) and/or pancolitis (94%) [14], suggesting that mild ileitis may be due to inflammation-induced incompetence of the ileocecal value with subsequent retrograde flow of colonic contents into the distal ileum (hence ‘backwash ileitis'), stasis due to inflammation-induced colonic hypomotility, or continuous extension of inflammation from the colon. The ileitis in UC is usually mild and consists of neutrophilic inflammation in the *lamina propria*, focal cryptitis/crypt abscesses and, less commonly, superficial mucosal erosions. Some cases may only show subtle features of mucosal injury, such as villous blunting and regenerative epithelial changes [14]. The presence of backwash ileitis in the colectomy specimen has no effect on the prevalence of pouch complications [14, 16]. While there is little information on backwash ileitis in initial endoscopic biopsies, one study found that 6% of patients with an ultimate diagnosis of pan-colonic UC had backwash ileitis in their initial biopsies, all of these with moderately to markedly active cecal chronic UC [15]. In our opinion, in biopsy material, it is reasonable to accept mild ileal inflammation as backwash ileitis, provided there is significant active cecal inflammation and there are no other histological features to suggest Crohn's disease.

#### Granulomas

**Figure 3.**
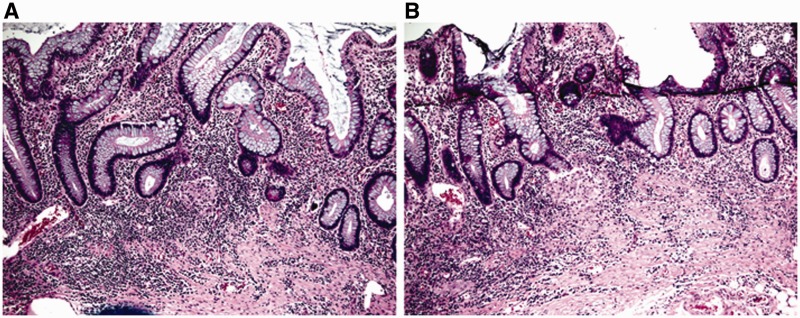
Ulcerative colitis, association of a cluster of histiocytes with damaged crypts confirmed by additional sections. A: proximity of histiocytes near a crypt (H&E stain; 100X). B: association of histiocytes with damaged crypts on deeper levels (H&E stain; 100X). The histiocytes contain pale foamy cytoplasm and represent a reaction to crypt rupture with extravasated mucin.

**Figure 4.**
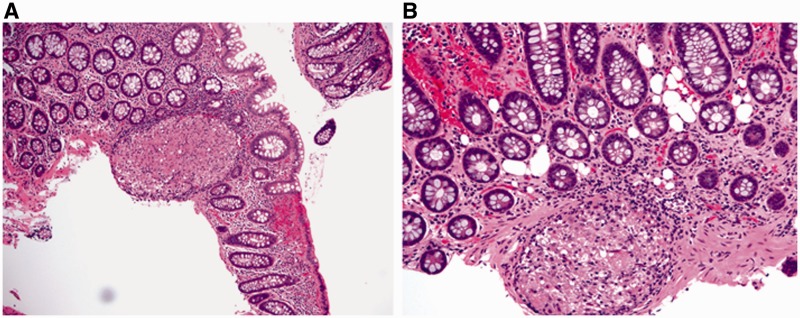
Discrete epithelioid granuloma in Crohn's disease. A: discrete epithelioid granuloma not associated with epithelial injury (H&E stain; 100X). B: discrete epithelioid granuloma in the *muscularis mucosa* (H&E stain; 200X). The histiocytes are epithelioid and contain abundant eosinophilic cytoplasm.

A granuloma is formed mostly by epithelioid histiocytes, with or without other cell types, including lymphocytes or multinucleated giant cells. It is important to recognize that not all granulomas are indicative of Crohn’s disease. In particular loosely formed microgranulomas, consisting of histiocytes and giant cells with pale foamy cytoplasm, may be seen in proximity to a damaged crypt or eroded surface; this should be viewed as a histiocytic response to extravasated mucin or fecal material, as a result of crypt injury or surface erosion (Figure 3A). Occasionally, the granuloma may be near—but not apparently associated with—a ruptured crypt on initial sections, but examining additional sections may confirm the association (Figure 3B). Well-formed epithelioid granulomas or isolated giant cells in the *lamina propria*, away from epithelial injury, should support a diagnosis of Crohn's disease (Figure 4) if associated with histological features of chronic colitis and if other etiologies for granulomas can be excluded [17].

### Features of treated UC

In the setting of medically treated UC, endoscopically or histologically discontinuous disease may be observed as a result of uneven healing [18–22]. The same process may also lead to absolute or relative rectal sparing. As patchiness of disease and rectal sparing mimic Crohn's disease and are commonly seen in treated UC, evaluation of disease distribution to sub-type IBD should not be attempted in this setting; rather, efforts should be directed toward identifying granulomas, superimposed infection, and dysplasia.

### Evaluation of colectomy specimens

In resection specimens, UC typically shows diffuse continuous mucosal disease (including the rectum), in the absence of fissuring ulcers, fistula tracts, transmural inflammation, small bowel involvement, or granulomas. However, in certain clinical settings, atypical features may be observed in colectomy specimens for UC. These atypical features include discontinuous disease and rectal sparing in medically treated or fulminant UC, and deep fissuring ulceration in fulminant UC.

### Discontinuous disease in colectomy specimens

In colectomy specimens, there are several circumstances in which UC may manifest a discontinuous pattern of disease, leading to a mis-diagnosis of Crohn's disease. These include: (i) mucosal healing in cases under treatment with either topical or oral agents, (ii) cecal and/or ascending colitis in patients with left-sided UC, (iii) appendiceal inflammation in patients with subtotal or left-sided colitis, (iv) patients with primary sclerosing cholangitis, and (v) fulminant colitis.

### Rectal sparing in colectomy specimens

UC classically involves the rectum, particularly in adult patients. Rectal sparing is seen in about 32% of cases by endoscopic examination and 30% by histological examination of biopsy specimens [22]. However, in colectomy specimens, only 5.4% of cases showed rectal sparing and all of these were considered ‘relative' [22]. These findings suggest that rectal sparing on biopsy material should not be interpreted as definite evidence of Crohn’s disease, and that assessment of colectomy specimens provides more accurate information on the status of the rectum.

### Fulminant UC in colectomy specimens

**Figure 5.**
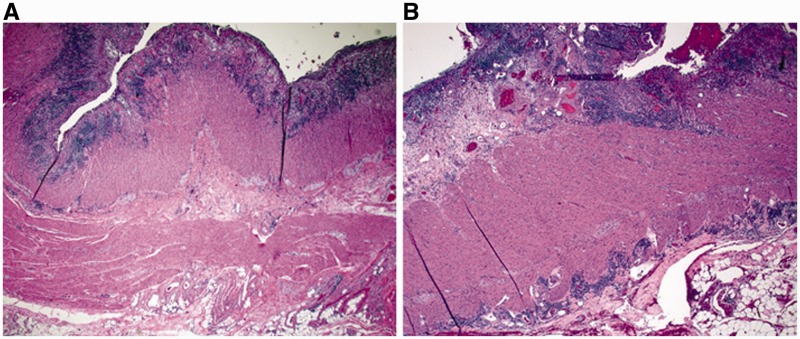
Histological features of fulminant colitis. Fissuring ulceration (A: H&E stain; 20X) and focal transmural inflammation near deeply ulcerated areas (B: H&E stain; 20X) are common findings.

Fulminant colitis is defined as severe, acute inflammation of the colon with associated systemic toxicity [23]. Most cases (89%) of fulminant colitis represent IBD, with the remainder relating to ischemia or infection, among other etiologies [13]. Macroscopic features such as dilation, skip lesions, rectal sparing, linear ulcers, terminal ileal disease, pseudopolyps, and creeping fat are poor discriminators of UC and Crohn's disease in setting of fulminant colitis. In particular, linear and/or fissuring ulcers (Figure 5A) and focal transmural inflammation near deeply ulcerated areas (Figure 5B) are commonly seen in the setting of fulminant UC and should not be considered to be diagnostic of Crohn's disease or ‘indeterminate' colitis. Granulomas and transmural lymphoid aggregates (away from areas of ulceration) are the two most specific indicators of Crohn's disease in this setting [13].

### Indeterminate colitis in colectomy specimens

Restorative proctocolectomy with ileal pouch-anal anastomosis (IPAA) has become the treatment of choice for UC patients requiring surgery for medically refractory disease or neoplastic complications. As IPAA is contra-indicated in patients with Crohn's disease because of high rates of severe complications and poor pouch outcomes, distinction of UC from Crohn's disease is essential. While most IBD cases can be correctly diagnosed in colectomy specimens, in approximately 5% of cases, the pathologist cannot establish a definite diagnosis of UC or Crohn’s disease for a number of reasons, including insufficient clinical-endoscopic-radiographic data, prominent overlapping features between these two diseases, or unfamiliarity with—or unwillingness to accept—atypical features of UC. In 1978, the concept of indeterminate colitis was introduced to describe cases in which colectomy had been performed for IBD, but a definitive diagnosis of UC *vs* Crohn's disease was not possible [24], often in the setting of steroid therapy or in cases of fulminant colitis, where specific features of either UC or Crohn’s disease have not fully manifested due to rapidly progressive disease. Indeterminate colitis should be considered as a provisional pathological diagnosis, as many cases may be more specifically categorized on the basis of clinical follow-up. In one series, approximately 80% of indeterminate colitis cases could be definitively categorized as UC or Crohn's disease within eight years [25]. Indeterminate colitis should not be used for biopsy cases of IBD with features equivocal for UC *vs* Crohn's disease, instead, these cases may be better termed “IBD, unclassified” or “equivocal/non-specific IBD” [26].

When indeterminate colitis is defined by the pathological findings in the resected colon as originally suggested, with features overlapping between UC and Crohn's disease, the incidence of pouch complications will lie between UC and Crohn's disease. However, if indeterminate colitis is defined as colitis showing features not classic for either UC or Crohn's disease, the incidence of complications and pouch failure will not differ from those in UC [27, 28]. Regardless of the exact definition of indeterminate colitis, if there are no pathological stigmata of Crohn’s disease in the biopsy and colectomy, or clinical evidence of Crohn's disease, patients with indeterminate colitis should not be denied an IPAA procedure [29, 30].

## UPPER GASTROINTESTINAL TRACT MANIFESTATIONS

While UC is traditionally considered to be a disease of the colon, inflammation of the upper gastro-intestinal (GI) tract has been described in a small proportion of UC patients. Gastric findings in UC patients include superficial plasmacytosis, focal gastritis, and basal mixed inflammation [31]. Duodenal pathology in UC includes diffuse chronic active duodenitis [31, 32] and intra-epithelial lymphocytosis, with or without partial villous atrophy. None of these patterns of upper GI inflammation are diagnostically specific for UC; in the absence of granulomas, the presence of upper GI inflammation in IBD patients should not necessarily be equated to Crohn’s disease.

## DIFFERENTIAL DIAGNOSES

### Crohn's disease

Crohn's disease remains the most difficult and critical differential diagnosis of UC and must be ruled out, based on a combination of clinical, radiographic, and histological features. The most suitable specimens for such a distinction are the first set of colonoscopic biopsies obtained prior to medical treatment and the colectomy specimen. If the first set of biopsies shows diffuse, chronic, active colitis involving the rectum and a variable length of colon in a continuous fashion, without granulomas or ileitis, the diagnosis is most likely UC if other etiologies can be excluded clinically. The presence of discrete epithelioid granulomas, unassociated with crypt rupture or foreign material, would favor Crohn's disease over UC. The presence of mild active ileitis, in the setting of pancolitis with marked right colonic inflammation, would be considered as backwash ileitis in UC. Similarly, involvement of the cecum and/or ascending colon in left-sided chronic active colitis should not be viewed as a skip lesion indicative of Crohn's disease. In colectomy specimens, relative (but not absolute) rectal sparing and segmental sparing (up to one section) are allowed in UC if the colitis is otherwise diffuse and superficial, without transmural inflammation and granulomas. The most difficult differential diagnosis is the recently described ‘ulcerative colitis-like Crohn's disease'. This refers to Crohn's disease limited to the colonic mucosa, without mural involvement in the form of fissuring ulcers, transmural lymphoid aggregates, sinus tracts, or fistulas [33]. This variant of Crohn’s disease can only be diagnosed by identifying the following features: segmental disease, creeping fat, absolute skip lesions, granulomas in the colectomy specimen, or clinical evidence of peri-anal disease [33].

### Infectious colitis and cord colitis

**Figure 6.**
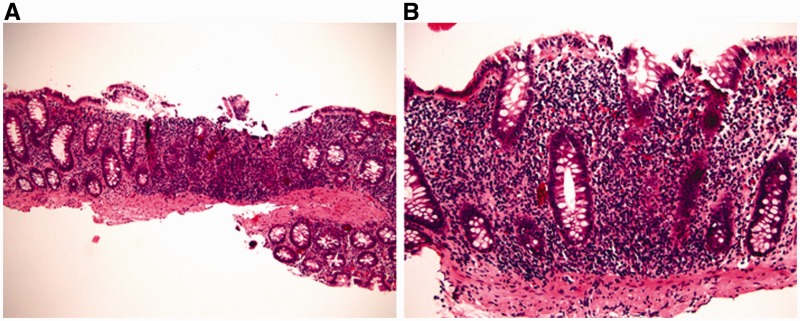
Infectious colitis typically shows acute colitis without architectural distortion (A: H&E stain; 100X. B: H&E stain; 200X).

**Figure 7.**
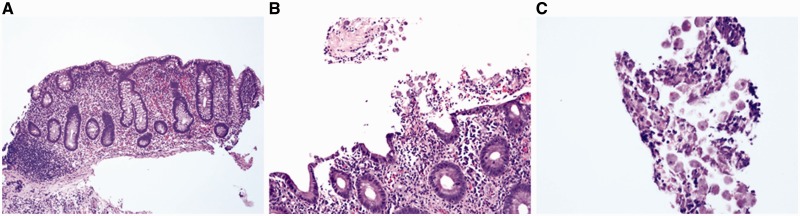
Amoebic colitis may mimic ulcerative colitis by a manifestation of chronic active colitis with crypt distortion, mild basal lymphoplasmatocysis (A: H&E stain; 100X), and epithelial injury (B: H&E stain; 200X). However, trophozoites of e*ntamoeba histolytica* are evident on high magnification (C: H&E stain; 400X).

Infectious colitis—due to a variety of agents—may clinically mimic UC. However, most cases of infectious colitis demonstrate a histological pattern of acute colitis, which may be diffuse, patchy, or focal, without evidence of chronicity, as evidenced by architectural distortion [34, 35] (Figure 6). Less commonly, chronic infectious colitis may produce a histological pattern of chronic active colitis resembling IBD. Most cases of infectious colitis with the chronic active colitis pattern have no specific diagnostic features on histological examination; in such cases, knowledge of the clinical history (particularly immunocompromised status) and correlation with serologic studies or stool cultures are required for diagnosis. However, an important exception is amoebic colitis, in which trophozoites of e*ntamoeba histolytica* may be observed in biopsy material showing features of chronic active colitis (Figure 7). Amoebae should always be excluded in biopsies of suspected IBD, as these organisms may not be identified by routine stool studies and because immunosuppressive therapy for presumed IBD may result in fulminant amoebic colitis with perforation [36].

**Figure 8.**
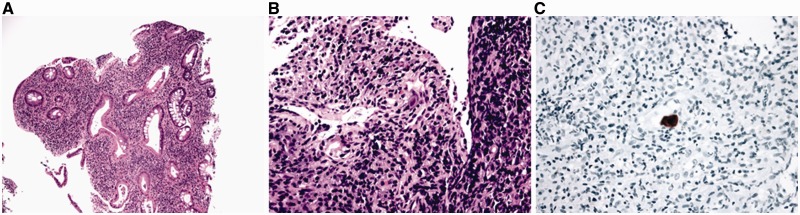
Cytomegaloviral (CMV) infection superimposed on ulcerative colitis. A: typical features of chronic active colitis (H&E stain; 100X). B: a CMV inclusion on H&E stain (H&E stain, 400X). C: a CMV inclusion identified by immunohistochemistry (immunoperoxidase stain; 400X).

As many types of infection can occur on a background of established IBD, all cases of IBD with exacerbation should be tested for the possibility of superimposed infection, including cytomegalovirus (CMV), *campylobacter* and *clostridium difficile*, among others. CMV is most commonly sought in acute exacerbations or in steroid-refractory disease and, in this setting, the diagnostic viral inclusions may be identified on routine stains (Figures 8A and B). However, if no CMV inclusions are identified on routine examination, immunohistochemical stains should be considered, as they are of superior diagnostic sensitivity to conventional hematoxylin and eosin stains (Figure 8C) [37].

A recently described culture-negative, antibiotic-responsive cord colitis syndrome in patients with cord blood stem cell transplants may exhibit features of chronic active colitis [38]. Unlike UC, basal plasmacytosis and substantial architectural distortion are usually not prominent in cord colitis. In addition, clinical correlation and an excellent response to antibiotic treatment should confirm the diagnosis.

### Medication-induced colitis

**Figure 9.**
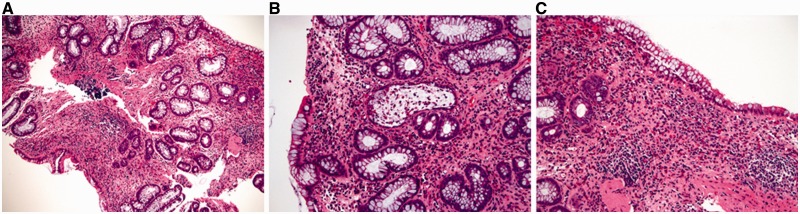
Mycophenolate-associated colonic injury may mimic ulcerative colitis by causing significant architectural abnormalities (A: H&E stain; 100X). However, the presence of abundant crypt apoptotic bodies (B: H&E stain; 200X) and prominent eosinophilic inflammation in the *lamina propria* (C: H&E stain; 200X) would favor mycophenolate colitis in the appropriate clinical setting.

Some forms of medication-induced colitis may demonstrate chronic active colitis, which may enter the differential diagnosis of UC. The key to correct diagnosis is to obtain a detailed medication history with relation to the onset of colitis and response to cessation of medication. Non-steroidal anti-inflammatory drugs (NSAIDs) are reported to cause GI injury in a significant number of patients; for example, they are reported to induce five different types of colitis: pseudomembranous colitis, eosinophilic colitis, collagenous colitis, *de novo* colitis, and reactivation of UC [39, 40]. In transplant patients, the immunosuppressant mycophenolate is associated with colonic injury, which may manifest as pancolitis and show IBD-like features in about 40% of cases (Figure 9) [41, 42]. Distinction of mycophenolate-induced colitis from UC may be histologically difficult; features favoring the former include crypt apoptosis and more prominent involvement in the right colon. However, rare cases of mycophenolate-induced colitis may evolve into IBD [41], indicating a need for clinical correlation and close follow-up.

### Chronic ischemia

Chronic ischemia may produce a pattern of chronic active enterocolitis and can present a difficult differential diagnosis, particularly in older patients and those with risk factors for ischemia. Chronic or recurrent ischemia may cause significant crypt distortion, Paneth cell metaplasia, and chronic active inflammation, all features which mimic IBD. However, atrophic and regenerative changes in the epithelium, hyalinization of the *lamina propria*, and the presence of microthrombi in the adjacent mucosa should suggest ischemia. Overall, in ischemia the chronic active inflammation is mild relative to the degree of epithelial injury. Further confounding this differential diagnosis, UC has been reported to cause a hypercoagulable state, particularly in patients who are genetically predisposed. In this setting, a superimposed arterial and venous thrombosis may occur, leading to severe steroid-refractory colitis [43].

### Diverticular disease-associated colitis

Chronic active colitis resembling UC may be seen in the setting of diverticulosis. Knowledge of the endoscopic impression of diverticulosis and the distribution of colitis is required for accurate diagnosis. Unlike UC, this diverticular colitis is confined to segments involved by diverticular disease, most commonly the sigmoid colon and, by definition, spares the rectum [44]. However, UC and diverticular colitis may in some cases represent overlapping entities, as a small subset of diverticular colitis patients have been reported as having progressed to typical rectosigmoid UC [44] and diverticular colitis may respond to medical therapy utilized for IBD [45].

## COLO-RECTAL NEOPLASIA IN PATIENTS WITH UC

Colo-rectal cancer (CRC) has been recognized as a leading cause of long-term mortality in patients with IBD and causes 8% of all deaths in patients with UC [46]. Regular colonoscopic surveillance examinations with biopsies have been used to identify dysplasia, the earliest recognizable precursor of CRC and the most reliable marker of cancer risk in this population. The optimal outcome of UC patients entering a surveillance program requires familiarity with the morphology and nomenclature of dysplasia, as well as knowledge of the limitations of histological evaluation for dysplasia.

### CRC in patients with UC

CRC in UC occurs in a background of chronically inflamed mucosa. The chronic colitis is usually appreciable on macroscopic examination. Compared with sporadic CRC, UC-associated CRC tends to be poorly delimited from the adjacent mucosa. Macroscopically, some cases mimic inflammatory strictures, fistula tracts, ulcers, and inflammatory polyps. Multifocal carcinoma is a common finding and is seen in up to 23% of cases [47, 48].

**Figure 10.**
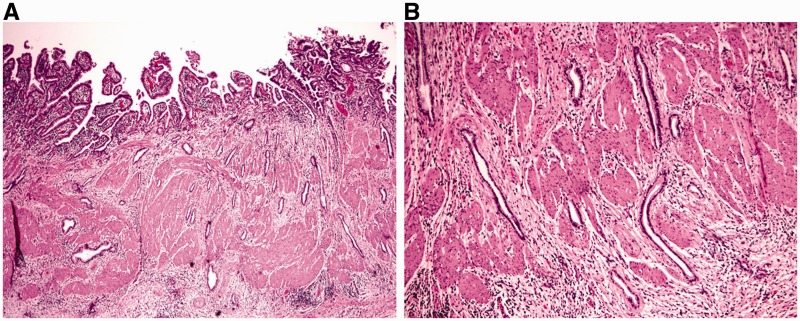
An example of extremely well-differentiated adenocarcinoma associated with ulcerative colitis (low-grade tubuloglandular adenocarcinoma). A: glands infiltrating through the *muscularis propria* (H&E stain; 40 X). B: bland histology of infiltrating glands (B: H&E stain; 100X).

Many cases of CRC arising in IBD histologically resemble sporadic adenocarcinomas. However, recent series have reported higher rates of mucinous differentiation (53% of cases), tumor heterogeneity (48%), and signet ring features (20% of cases) in UC-associated CRCs, compared with sporadic tumors. In addition, UC-associated CRCs are more often well-differentiated (about 34%) [48]. Low-grade tubuloglandular adenocarcinomas, extremely well-differentiated adenocarcinomas rarely encountered outside the setting of colitis, account for 11% of IBD-associated CRC (Figure 10) [49].

### Colo-rectal dysplasia in patients with UC

Dysplasia of the colorectum is defined as an unequivocal neoplastic alteration of the epithelium that remains confined within the basement membrane within which it originated [50]. While polypoid forms of UC-associated dysplasia have previously been described [51], in 1967 Morson and Pang showed that pre-cancerous changes in flat mucosa (flat dysplasia) detected by random rectal biopsies strongly predicted synchronous carcinomas in the remaining colon [52]. Dysplasia is not only a marker of synchronous invasive adenocarcinoma (prevalent carcinoma) but also a precursor, with the potential to progress to carcinoma (incident carcinoma). Since histological diagnosis of dysplasia was first proposed as an aid to cancer control in UC, it has remained the best predictor of neoplastic progression in this setting.

Riddell *et al.* [50] proposed a classification for the evaluation of colonic epithelial changes in IBD that consisted of three major categories: negative, indefinite, and positive for dysplasia [50], with ‘positive for dysplasia' further divided into low-grade (LGD) and high-grade (HGD). The diagnosis of dysplasia is based on the presence of a combination of microscopic features, including (i) architectural alterations exceeding those resulting from repair in chronic colitis, and (ii) cytological abnormalities, after excluding the possibility of inflammatory and reparative changes that may affect the colonic mucosa in chronic colitis.

### Epithelium negative for dysplasia

**Figure 11.**
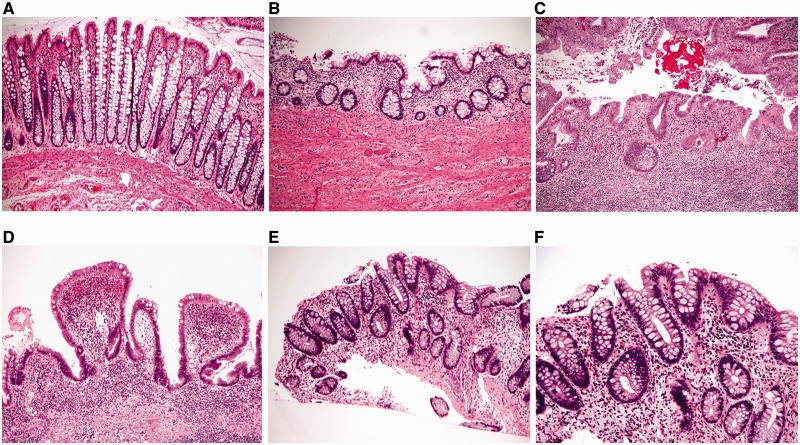
The category of mucosa negative for dysplasia includes normal mucosa (A: H&E stain; 100X), quiescent colitis (B: H&E stain; 100X), chronic active colitis (C: H&E stain; 100X; D: H&E stain; 100X), and the resolving phase of ulcerative colitis (E: H&E stain; 100X; F: H&E stain; 200X). In all scenarios, there are either no cytological atypia or none beyond that expected for reactive changes.

In normal colonic mucosa the crypts are straight tubular structures that are regularly distributed throughout the mucosa, arranged parallel to one another, and extend to touch the *muscularis mucosae*. Mononuclear inflammatory cells are present in the *lamina propria* but should not noticeably expand it. Neither neutrophilic inflammation nor cytological atypia are present (Figure 11A).

Quiescent (inactive) colitis refers to architectural abnormalities of chronic colitis in the absence of significant neutrophilic crypt injury. These changes include atrophy, irregularity and shortening of crypts, thickening of the *muscularis mucosae*, or metaplasia (Paneth cell or pyloric metaplasia) (Figure 11B).

The active and resolving phase of UC may pose diagnostic challenges in evaluating for dysplasia. In the active phase of UC, the damaged and regenerative epithelium may show loss of mucin, a feature also seen in dysplastic cells. However, the presence of chronic active inflammation, surface and crypt epithelial damage, and the lack of significant nuclear enlargement, hyperchromasia, and pleomorphism, should allow an interpretation of negative for dysplasia in this scenario (Figures 11C and D). The resolving or regenerative phase of UC is well described but may pose the greatest diagnostic challenge with respect to interpretation of dysplasia. In the resolving phase, there may no longer be neutrophilic crypt injury (including erosion or ulceration) and the crypts show varying degrees of nuclear enlargement, hyperchromasia, and nuclear stratification (Figures 11E and F). Mitotic activity may be brisk. However, the presence of at least partial surface maturation (i.e. the nuclear changes in the regenerative crypts do not extend on to the surface mucosa), and the increased inflammatory cells in the *lamina propria* are clues to regenerative changes. In difficult cases, knowledge that the patient has been recently treated for an acute flare may be helpful.

### Epithelial changes indefinite for dysplasia

This category refers to ambiguous epithelial alterations that cannot with certainty be classified either as negative or positive for dysplasia. There are a myriad histological patterns that may be categorized as indefinite for dysplasia. Most commonly, there are atypical cytological features in the setting of florid inflammation or ulceration; in this setting, it may be difficult to distinguish regenerative changes from low-grade or sometimes high-grade dysplasia. Another setting is the presence of severe nuclear abnormalities in the crypt bases when the surface mucosa cannot be evaluated. As the crypt bases constitute the regenerative compartment of intestinal mucosa, cytological changes in crypt bases must always be interpreted in comparison to the mucosal surface; if the cytological changes in the crypts do not involve surface epithelium, they are most likely reactive in nature. For this reason, in cases with cytological atypia involving the crypt bases, the absence of surface epithelium due to ulceration or mechanical denudation may result in an interpretation of indefinite for dysplasia. In addition, various cytological features that arise as effects of histological processing such as suboptimal embedding, tangential sectioning, and staining or fixation artifacts may lead to an interpretation of indefinite for dysplasia.

**Figure 12.**
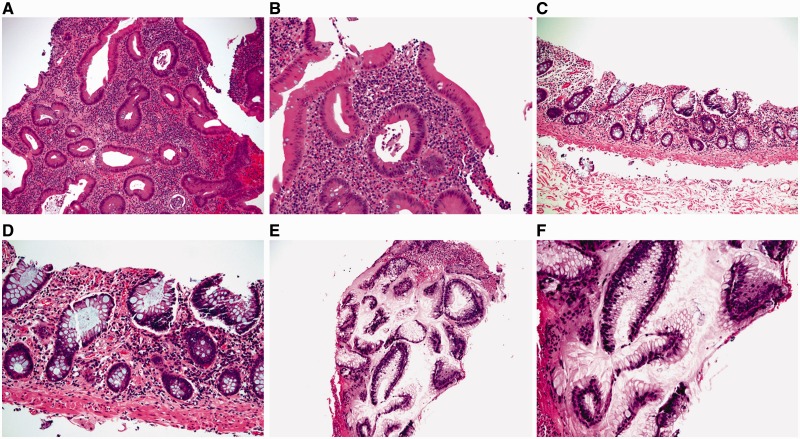
Epithelial changes indefinite for dysplasia, probably negative. A and B: there is slight nuclear enlargement and hyperchromasia in the presence of significant inflammation and nearby ulceration (A: H&E stain; 100X; B: H&E stain; 200X.) C and D: epithelial changes indefinite for dysplasia, unknown. This case demonstrates slight hyperchromasia and nuclear enlargement in the epithelium but there is no surface epithelium present for the evaluation of surface maturation (C: H&E stain; 100X; D: H&E stain; 200X). E and F: epithelial changes indefinite for dysplasia, probably positive. There are enlarged nuclei with hypechromasia and stratification in the detached epithelium (E: H&E stain; 100X; F: H&E stain; 200X).

Cases that are indefinite for dysplasia may be optionally subdivided into ‘probably negative', ‘unknown', and ‘probably positive' (Figure 12). These subdivisions can be particularly useful in patient management if the factors leading to the interpretation are clearly conveyed to the clinician.

### Epithelium positive for dysplasia

This category is subdivided into low-grade dysplasia (LGD) and high-grade dysplasia (HGD), based on the degree of architectural and cytological abnormalities [50], to imply the relative risk of a concurrent adenocarcinoma or progression to adenocarcinoma.

#### Positive for dysplasia: LGD

**Figure 13.**
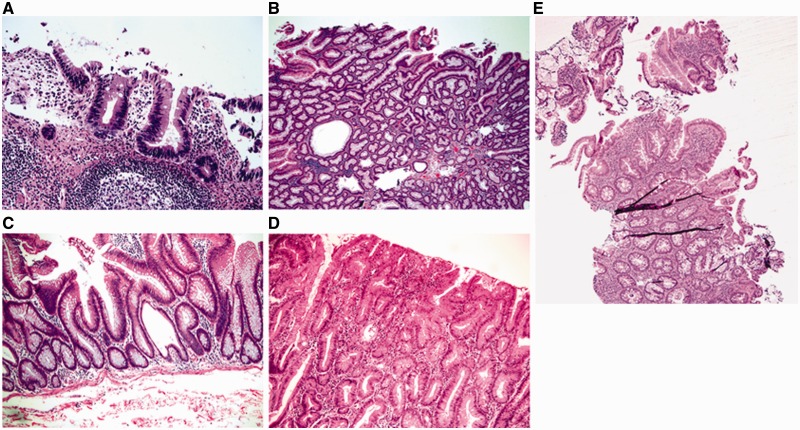
Low-grade dysplasia. A: flat low-grade dysplasia. This biopsy is from flat colonic mucosa from a patient with ulcerative colitis. The epithelium shows slightly hyperchromatic, stratified nuclei without surface maturation (H&E stain; 100X). B: polypoid fragment of low-grade dysplasia. The overall features are identical to those seen in colonic adenomas in non-colitic patients (H&E stain; 40X). The clinical significance and management of this type of dysplasia requires clinical and close endoscopic correlation. C: flat low-grade dysplasia with villous configuration (H&E stain; 100X). There is no significant pleomorphism or loss of polarity of the nuclei. D: flat low-grade dysplasia with pyloric gland features (H&E stain; 100X). There is uniform, monotonous proliferation of cuboidal cells without surface maturation. E: polypoid traditional serrated adenoma (TSA)-like low-grade dysplasia. Some of the epithelium is relatively eosinophilic and resembles that of sporadic TSAs (E: H&E stain; 40X).

Most biopsy specimens of LGD have crypts lined by epithelium with enlarged and hyperchromatic nuclei. Nuclear stratification is present but typically confined to the basal half of the cells (Figures 13A and B). The nuclei in LGD maintain normal polarity; that is, their long axes are perpendicular to the basement membrane. Most cases of LGD do not exhibit surface nuclear maturation: in other words, atypical nuclear features should involve both crypt and surface epithelium (Figures 13A–E).

#### Positive for dysplasia: HGD

**Figure 14.**
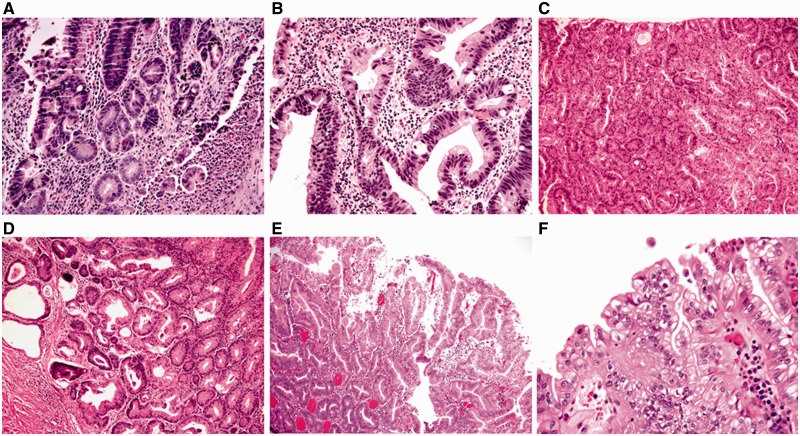
High-grade dysplasia. A: high-grade dysplasia manifested by proliferation of glands lined by round cells with enlarged nuclei. Some of the nuclei are hyperchromatic but some of them contain open chromatin (H&E stain; 400X). B: high-grade dysplasia characterized by marked pleomorphism (H&E stain; 400X). C and D: high-grade dysplasia with crowding glandular proliferation (C: H&E stain; 100X; D: H&E stain; 200X). E and F: high-grade dysplasia with architectural complexity and focal clear cell features (E: H&E stain; 100X; F: H&E stain; 400X).

In the originally proposed classification, the majority of HGD cases closely resemble adenomas in non-colitic patients with nuclear stratification extending to the superficial aspect of the epithelial cells. HGD exhibits enlarged nuclei with marked nuclear hyperchromasia, pleomorphism, and loss of nuclear polarity (Figures 14A and B). In addition to these nuclear features, HGD may exhibit increased architectural complexity, which may manifest as crowded or cribriform glands or villiform/papillary surface configuration (Figures 14 C–F).

### Special issues of dysplasia in patients with UC

#### Dysplasia identified in endoscopically apparent lesions in patients with UC

Sometimes, a diagnosis of dysplasia is made, based on biopsy material taken from an endoscopically evident polyp or mass; this is historically termed ‘dysplasia associated lesion or mass' (DALM) and considered a strong indication for colectomy because of a high risk of associated carcinoma [53]. Subsequently, the concept of DALM was subdivided into three categories, based on the endoscopic appearance and location of the polyp: (i) sporadic adenoma if the polyp resembles an adenoma both endoscopically and histologically and is located outside areas of histologically proven colitis; (ii) UC-associated adenoma-like polypoid dysplasia if the lesion resembles an adenoma both endoscopically and histologically and is located in the areas of colitis, and (iii) UC-associated non-adenoma-like dysplasia (‘true DALM') if the elevated or flat lesion is irregular and broadly based or forms a mass and is located in areas of colitis [54]. Isolated polypoid dysplastic lesions occurring outside areas of histologically proven colitis are considered as sporadic adenomas, as none of these patients developed flat dysplasia or adenocarcinoma during follow-up and complete polypectomy with routine surveillance colonoscopy should be adequate [55]. UC-associated adenoma-like dysplasia exhibits genotypic abnormalities overlapping with sporadic adenomas (loss of heterozygosity for 3 p, p16, and APC), suggesting that these two entities are related [54]. When not associated with flat dysplasia or carcinoma, polypectomy with surveillance at a shortened interval may be an adequate option for these UC-associated adenoma-like lesions [56]. As UC-associated non-adenoma-like DALMs have a different molecular genotype from adenoma-like polypoid dysplasia and sporadic adenomas [54] and a high risk of associated carcinoma, they should be treated with colectomy after the diagnosis of dysplasia is confirmed [50].

Clinical correlation with the extent of colitis and location and endoscopic appearance of the polyp are essential for proper classification. It cannot be over-emphasised that the endoscopic impression of resectability is the key factor in determining appropriate management for visible dysplastic lesions in UC, as it is extremely difficult, if not impossible, to distinguish these entities on the basis of histological features alone. In addition, biopsy of the mucosa surrounding the polyp and attention to the epithelium of the polyp stalk may be useful in determining appropriate management. Regardless of whether an endoscopically resectable lesion represents polypoid IBD-associated dysplasia or a sporadic adenoma, this distinction is of little consequence as in most cases these may be adequately managed with complete polypectomy and close endoscopic surveillance. On the other hand, endoscopically unresectable lesions pose a high risk for adenocarcinoma and are typically managed with colectomy.

### Difficulties in diagnosis of UC-associated dysplasia

As UC-associated dysplasia may occur in endoscopically normal mucosa, extensive biopsy sampling is necessary to confidently exclude the possibility of dysplasia. In order to exclude dysplasia with 90% confidence, a minimum of 33 well-oriented jumbo forceps biopsies are required; 56 such biopsies are required to achieve a 95% level of confidence [57]. This is the rationale for surveillance programs including periodic colonoscopic examination with protocol biopsies (four quadrant biopsies every 10 cm) to detect dysplasia, which has now become the standard of care for surveillance in IBD patients [47].

In addition to sampling error, histological evaluation of dysplasia suffers from problems of diagnostic reproducibility. Few studies have evaluated inter-observer agreement in the diagnosis of UC-associated dysplasia [50, 58 ,59], none of which assessed inter-observer agreement in the context of clinical outcome, the ultimate standard of diagnosis.

The interpretation of indefinite for dysplasia has the highest level of disagreement, followed by biopsies with LGD [50, 58, 59]; these findings are not surprising, given the frequent difficulty in distinguishing inflammation-associated regenerative changes from LGD. Reproducibility and agreement are best at the extremes of diagnosis: negative for dysplasia [50, 59] and HGD [50, 58, 59]. While it is fortunate that inter-observer variability is less pronounced for HGD, given the indication for definitive management of HGD, there is nevertheless only moderate inter-observer agreement among both gastro-intestinal pathologists [58, 59] and general pathologists [58] in respect of this diagnosis. It is our opinion that interpretations of LGD and HGD should be confirmed by a gastro-intestinal pathologist prior to any invasive intervention.

### Clinical management of UC patients in surveillance program

Histological interpretation of surveillance biopsies plays an essential role in clinical management. There is unanimous agreement in the literature that the detection of flat HGD or a DALM with any degree of dysplasia carries a sufficiently high risk (about 40% for HGD and 30% for DALMs) of prevalent CRC or short-term and high-risk progression to CRC (25%) to warrant immediate colectomy [60, 61]. The natural history of LGD is more controversial but, in two studies, LGD was associated with a 20% risk of prevalent CRC in patients who underwent immediate colectomy or colectomy within 6 months and 14.5–19.4% risk of progressing to CRC in patients who continued on surveillance alone [61, 62]. While outcome data are scarce for UC with changes indefinite for dysplasia, this diagnosis is associated with significant risk of prevalent HGD (5 of 22 cases: 27.3%) and significant progression rates to dysplasia (3.2 cases/100 person-years) and advanced neoplasia (1.5 cases/100 person-years) [63], suggesting that UC patients with this finding warrant close follow-up.

**Conflict of interest:** none declared.
